# PR-104 a bioreductive pre-prodrug combined with gemcitabine or docetaxel in a phase Ib study of patients with advanced solid tumours

**DOI:** 10.1186/1471-2407-12-496

**Published:** 2012-10-25

**Authors:** Mark J McKeage, Michael B Jameson, Ramesh K Ramanathan, Joseph Rajendran, Yongchuan Gu, William R Wilson, Teresa J Melink, N Simon Tchekmedyian

**Affiliations:** 1 The University of Auckland, Auckland, New Zealand; 2 Waikato Hospital, Hamilton, Waikato, New Zealand; 3 Virginia G Piper Cancer Center & TGEN, Scottsdale, AZ, USA; 4 University of Washington, Seattle, WA, USA; 5 Proacta Inc, San Diego, CA, USA; 6 Pacific Shores Medical Group, Long Beach, CA, USA; 7 Department of Pharmacology and Clinical Pharmacology and the Auckland Cancer Society Research Centre, School of Medical Sciences, Faculty of Medical and Health Sciences, The University of Auckland, Private Bag 92019, 85 Park Road Grafton, Auckland, 1142, New Zealand

## Abstract

**Background:**

The purpose of this phase Ib clinical trial was to determine the maximum tolerated dose (MTD) of PR-104 a bioreductive pre-prodrug given in combination with gemcitabine or docetaxel in patients with advanced solid tumours.

**Methods:**

PR-104 was administered as a one-hour intravenous infusion combined with docetaxel 60 to 75 mg/m^2^ on day one given with or without granulocyte colony stimulating factor (G-CSF) on day two or administrated with gemcitabine 800 mg/m^2^ on days one and eight, of a 21-day treatment cycle. Patients were assigned to one of ten PR-104 dose-levels ranging from 140 to 1100 mg/m^2^ and to one of four combination groups. Pharmacokinetic studies were scheduled for cycle one day one and ^18^F fluoromisonidazole (FMISO) positron emission tomography hypoxia imaging at baseline and after two treatment cycles.

**Results:**

Forty two patients (23 females and 19 males) were enrolled with ages ranging from 27 to 85 years and a wide range of advanced solid tumours. The MTD of PR-104 was 140 mg/m^2^ when combined with gemcitabine, 200 mg/m^2^ when combined with docetaxel 60 mg/m^2^, 770 mg/m^2^ when combined with docetaxel 60 mg/m^2^ plus G-CSF and ≥770 mg/m^2^ when combined with docetaxel 75 mg/m^2^ plus G-CSF. Dose-limiting toxicity (DLT) across all four combination settings included thrombocytopenia, neutropenic fever and fatigue. Other common grade three or four toxicities included neutropenia, anaemia and leukopenia. Four patients had partial tumour response. Eleven of 17 patients undergoing FMISO scans showed tumour hypoxia at baseline. Plasma pharmacokinetics of PR-104, its metabolites (alcohol PR-104A, glucuronide PR-104G, hydroxylamine PR-104H, amine PR-104M and semi-mustard PR-104S1), docetaxel and gemcitabine were similar to that of their single agents.

**Conclusions:**

Combination of PR-104 with docetaxel or gemcitabine caused dose-limiting and severe myelotoxicity, but prophylactic G-CSF allowed PR-104 dose escalation with docetaxel. Dose-limiting thrombocytopenia prohibited further evaluation of the PR104-gemcitabine combination. A recommended dose was identified for phase II trials of PR-104 of 770 mg/m^2^ combined with docetaxel 60 to 75 mg/m^2^ both given on day one of a 21-day treatment cycle supported by prophylactic G-CSF (NCT00459836).

## Background

**Figure 1:**
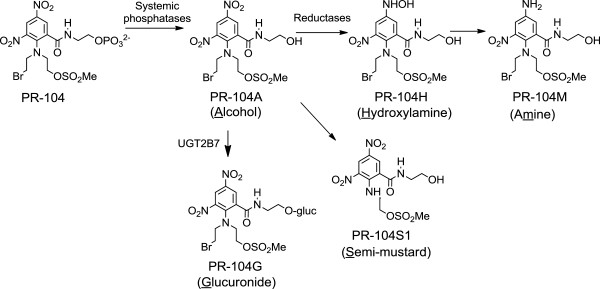
**Chemical structures of PR-104 and its biotransformation products.** PR-104 undergoes rapid hydrolysis by systemic phosphatases to PR-104A that becomes activated by NADPH-cytochrome P450 oxidoreductases and other one-electron reductases in hypoxia, or in oxic conditions by AKR1C3, to reactive nitrogen mustards that crosslink DNA causing tumour cytotoxicity. PR-104 is metabolically deactivated by glucuronidation or N-dealkylation

New approaches to cancer treatment are needed urgently. Tumour hypoxia is a common feature of human cancer, and its presence is associated with poor patient prognosis and tumour resistance to radiotherapy and chemotherapy [1,2]. In addition, aldoketoreductase 1C3 (AKR1C3) may be over-expressed by many human cancers [3]. PR-104 is a phosphate ester dinitrobenzamide mustard precursor of the prodrug PR-104A that is designed to become activated into cytotoxic nitrogen mustards in tumour regions that are either hypoxic or express AKR1C3 (Figure 1). After rapid hydrolysis of PR-104 to PR-104A by systemic phosphatases, PR-104A becomes activated by NADPH-cytochrome P450 oxidoreductases and other one-electron reductases in hypoxia, or under oxic conditions by AKR1C3, to reactive nitrogen mustards (hydroxylamine PR-104H and amine PR-104M) that crosslink DNA causing tumour cytotoxicity [3-6]. Previous single agent phase I clinical trials of PR-104 given as a one hour intravenous infusion identified thrombocytopenia, neutropenia, infection and fatigue as its dose-limiting toxicities (DLTs) and a maximum tolerated dose (MTD) of 1100 mg/m^2^ given once every 21 days [7] or 675 mg/m2 given on days 1, 8 and 15 every 28 days [8]. Preclinical *in vivo* combination antitumour studies showed PR-104 to have additive or super-additive efficacy in combination with several established anticancer drugs, including docetaxel and gemcitabine [5]. Docetaxel and gemcitabine are approved agents for the treatment of a wide range of human malignancies, including breast, head and neck, non-small cell lung, ovarian, pancreatic and prostate cancer [9,10], but their clinical efficacy may be limited by their inability to effectively treat hypoxic areas of tumours [11,12]. These considerations led us to undertake this phase Ib, multicentre, open label, serial cohort, non-randomized, uncontrolled trial of PR-104 given in combination with docetaxel or gemcitabine in patients with advanced solid tumours. The primary objective was to determine the MTD of PR-104 given in combination with docetaxel or gemcitabine. Secondary objectives were to evaluate the safety and tolerability, antitumour activity and pharmacokinetics of PR104 combined with docetaxel or gemcitabine. An ancillary objective was to undertake a clinical assessment of tumour hypoxia with ^18^F-fluoro-misonidazole (F-MISO) positron emission tomography (PET) scanning in the context of a multicentre phase I oncology clinical trial. This imaging technique noninvasively demonstrates anatomical regions of high F-MISO uptake and hypoxia [13], whose detection may be predictive of the therapeutic efficacy of hypoxia-activated antitumour therapies. 

## Methods

Thus study was conducted after its approval by relevant ethics committee (Northern X Regional Ethics Committee for New Zealand clinical sites), institution review boards (Western Institutional Review Board for USA clinical sites), regulatory authorities and other institutional committees, and its registration with *ClinicalTrials.gov* (NCT00459836).

### Patient eligibility

Selection criteria for patient enrolment into this study included age 18 years or more; histologically-confirmed malignancy for which treatment with gemcitabine or docetaxel in combination with an investigational agent was considered clinically appropriate; measurable or evaluable disease; ECOG Performance Status of 0 or 1; ability to provide written informed consent; no or stable dose of systemic steroid for at least two weeks; adequate bone marrow function (absolute neutrophil count ≥ 1.5 x 10^9^/L, platelet count ≥ 100 x 10^9^/L, haemoglobin level ≥ 90 g/L not maintained by red blood cell transfusion, prothrombin and activated partial thromboplastin times ≤ 1.1 x upper limit of normal (ULN)); adequate liver function (serum bilirubin within normal limits, ALT and AST ≤ 2.5 x ULN), and serum creatinine ≤ 1.5 x ULN. Exclusion criteria included: licensed or investigational anti-cancer therapy (including radiotherapy but excluding androgen deprivation therapy) within four weeks; nitrosoureas or mitomycin C within six weeks; prior radiotherapy to more than 25% of bone marrow; prior high-dose chemotherapy; prior receipt of more than three chemotherapy regimens; pregnancy, breast feeding or plans for becoming pregnant during the study; unwillingness to use effective contraception during the study and for 30 days following the last dose of study medication; other medical disorder or laboratory finding that in the opinion of the investigator compromised subject safety; less than four weeks since major surgery, or; HIV, hepatitis B surface antigen or hepatitis C positivity with abnormal liver function tests.

### Study design

This was a multicentre phase Ib, multiple-arm, nonrandomized, open label, uncontrolled, serial cohort, dose-escalation study evaluating PR-104 in combination with gemcitabine or docetaxel. A conventional three-plus-three phase I study design was used to establish the MTD of these PR-104 chemotherapy combinations.

### Drug administration and dose escalation scheme

A lyophilized cake of 400 mg of PR-104 was reconstituted with two mL of water for injection, further diluted in 250 mL of 5% dextrose in water and administered as an intravenous infusion over one hour on day one of a 21 day treatment cycle with docetaxel and on days one and eight of a 21 day treatment cycle with gemcitabine. The PR-104 starting dose-level was determined from its phase Ia clinical trials [7,8]. The PR-104 dose-level was escalated or de-escalated by 0.5-, 1.25-, 1.5- or 2.0-times according to the number of subjects with DLT at the previous dose-level. The safety committee could modify the dose-escalation plan by adding patients, intermediate dose-levels and supportive care therapies or by adjusting the combination agent dose, as appropriate according to the accruing safety data. Docetaxel (60 or 75mg/m^2^) was administered intravenously over 1 h following the PR-104 infusion on day one of every 21 day treatment cycle. Gemcitabine (800 mg/m^2^) was administered as a 30 min intravenous infusion immediately following the one hour PR-104 infusions both given on days one and eight of every 21 day treatment cycle. For the two combination groups exploring PR104 and docetaxel with prophylactic G-CSF, G-CSF (either Neupogen® or Neulasta®) was administered as a subcutaneous injection beginning on day 2 at the approved dose and schedule for Neupogen® or Neulasta®. Prophylactic anti-emetics were administered to all study patients according to local institutional guidelines.

### Definition of DLT and MTD

For this study, toxicity was assessed according to the National Cancer Institute Common Toxicity Criteria for Adverse Events (CTCAE version 3.0). DLT was assessed during the first three weeks following day one of cycle one. DLT was defined as any one of the following: grade four thrombocytopenia (platelets < 25 x 10^9^/L) of any duration; other grade four haematological toxicity that lasted for five days or more (haemoglobin < 65 g/L, neutrophils < 0.5 x 10^9^/L); non-haematological toxicity ≥ grade three despite appropriate treatment; neutropenic fever; grade two or higher neurotoxicity that lasted one week or more; any toxicity of grade two or higher that had not resolved within two weeks of the end of cycle one (except grade two alopecia). The MTD was defined as a dose level at which one or fewer of a cohort of six patients exhibited DLT, that was immediately below a dose-level where two or more of a cohort of up to six subjects had demonstrated DLT.

### Patient evaluation and follow-up

Following written informed consent, baseline evaluations included a history and physical examination, assessment of performance status, concomitant medications, complete blood count (CBC), blood chemistry profile, coagulation studies (INR and APPT), urinalysis, pregnancy test and serum tumour markers. Vital signs and electrocardiogram were taken before, during and after the administration of the first dose of PR-104 combination therapy. Weekly assessments on study included interim medical history, symptom-directed physical examination, patient performance status, laboratory investigations (CBC, coagulation studies, serum chemistry and urinalysis), inter-current adverse events and concomitant medication use. Disease was assessed by computed tomography or magnetic resonance scans within one month prior to cycle one day one and repeated once every two treatment cycles. Tumour response to treatment was assessed using Response Evaluation Criteria in Solid Tumours (RECIST) criteria version 1.0 [14].

### ^18^F-MISO PET Imaging

F-MISO PET scans were scheduled to be performed at baseline and after day 15 of the second treatment cycle. F-MISO was prepared in ≤ 10mL of 95% isotonic saline and 5% ethanol and given at a dose of 0.1mCi/kg, that did not exceed 10 mCi) at a specific activity of >125 Ci/mmol at the injection time. PET scanning began 90 to 120 min after intravenous administration of F-MISO with venous blood sampling at 5, 10 and 15 min after the commencement of the scan. Tumour-to-blood ratios were calculated and those ≥1.2 were regarded as indicating significant tumour hypoxia.

### Pharmacokinetic analyses

Blood samples for pharmacokinetic (PK) analyses were collected on cycle one day one into ETDA vacutainer tubes before PR-104 infusion, during the infusion (45 min after the commencement of the infusion), immediately after completion of the infusion, and at 5, 10, 20, 30, 45, 60, 120 and 240 min and 24 h after the completion of PR-104 infusion. Blood samples were centrifuged for five minutes to prepare plasma. Plasma was then immediately deproteinised by addition of nine volumes of methanol:ammonium acetate: acetic acid (1000:3.5:0.2 v/w/v) and stored at −70°C until analysis. To evaluate plasma concentrations of PR-104, PR-104A and its major metabolites (Figure 1), extracts of plasma were assayed by validated ultra high-performance liquid chromatography methods [15] using triple quadrupole mass spectrometric detection with tetradeuterated internal standards [16]. Blood samples for PK analyses of gemcitabine were collected on cycle one day one prior to gemcitabine infusion, immediately after completion of the infusion and at 20, 40, 60, 90, 120 and 240 min and 8 and 24 h after completion of gemcitabine infusion. Concentrations of gemcitabine and its inactive metabolite difluorodeoxyuridine were determined in human plasma samples by HPLC and MS/MS detection using 2-deoxyuridine as an internal standard. The correlation coefficients for the calibration curves were r^2^ > 0.99. Assay accuracy and precision ranged from 92.3 to 108.3% and from 2.2 to 14%, respectively. The lower limit of quantitation for gemcitabine and difluorodeoxyuridine was 50 ng/ml and 500 ng/ml, respectively. Blood samples for PK analyses of docetaxel were collected on cycle one day 1 prior to docetaxel infusion, during the infusion (40 min after commencement of the infusion) and immediately after completion of the infusion, and at 20, 40, 60, 90 120 and 240 min and 8 and 24 h after completion of docetaxel infusion. Concentrations of docetaxel were determined in human plasma samples treated by solvent extraction with hexane followed by HPLC and MS/MS detection using paclitaxel as an internal standard. The correlation coefficients for the calibration curves were r^2^ > 0.99. Assay accuracy was ± 5.4% of the expected value and precision ranged from 6.46 to 8.6%. The lower limit of quantitation for docetaxel was 0.5 ng/ml. Non-compartmental pharmacokinetic analyses using WinNonLin (v4.0.1) or PK Solver (version 2.0), and actual infusion times and doses, were used to generate pharmacokinetic parameters including the area under the plasma concentration time curve extrapolated to infinity (AUC_0-inf_) or to the last sample time-point (AUC_0-t_) and elimination half-life (t_1/2_).

### Statistics

Data were analysed using descriptive statistics including the median, range and proportion, and mean and standard deviation for normally distributed data. Cohorts of up to six patients at each PR-104 dose-level were considered adequate for defining the DLT and MTD.

## Results

### Patient characteristics

This phase Ib clinical trial enrolled a total of 42 patients that included 23 females and 19 males whose ages ranged from 27 to 85 years (Table 1). They had cancers of the lung (10 patients), gastrointestinal tract (7 patients in total including 4 patients with pancreatic cancer and one each with gastric, colorectal or oesophageal cancer), genitourinary tract (7 patients), prostate (4 patients), melanoma (3 patients), sarcoma (2 patients) or other tumour sites (8 patients). Most had received prior chemotherapy usually with one or two, but never more than three, prior regimens. Five of 42 patients (12%) had been previously exposed to the standard chemotherapy agent they were given in combination with PR-104 in the trial.

**Table 1:** Patient characteristics

**Characteristic**		**Number of Patients (%) (n=42)**
Gender		
	Female	23 (55%)
	Male	19 (45%)
Age (Years)		
	Median (range)	60 (27–85)
Ethnicity		
	Caucasian	34 (81%)
	Other	8 (19%)
ECOG performance status		
	Median (range)	1 (0–1)
Tumour type		
	NSCLC	10 (24%)
	Gastrointestinal	7 (17%)
	Genitourinary	4 (10%)
	Prostate	4 (10%)
	Head and neck	3 (7%)
	Melanoma	3 (7%)
	Sarcoma	3 (7%)
	Other	8 (19%)
Number of prior chemotherapy regimens		
	Median (range)	1 (0–3)

### Study treatment assignment

Serial patient cohorts, comprising of three or six subjects in each, were assigned to one of ten different PR-104 dose-levels ranging from 140 to 1100 mg/m^2^ given as a one hour intravenous infusion on day one (and day eight when combined with gemcitabine) of a 21 day treatment cycle (Table 2). In addition, patients were assigned to one of four different PR-104 combination treatment groups (Table 2). In Group A, a total of nine patients were given PR-104 with gemcitabine 800 mg/m^2^ on days one and eight of a 21 day treatment cycle. In Group B, a total of six patients were given PR-104 with docetaxel 60 mg/m^2^ on day one of a 21 day treatment cycle. In Group C a total of 21 patients were given the same treatment as Group B except with the addition of G-CSF from day two of each treatment cycle. In Group D, a total of six patients limited to one prior chemotherapy regimen were given the same treatment as Group C except that the dose of docetaxel was increased to 75 mg/m^2^.

**Table 2:** PR104 starting and maximal tolerated doses with its combination agents, their doses and administration schedules

**Group**	**PR104 dose (mg/m**^**2**^**)**		**Combination Agents**		**Schedule**
	**Starting**	**Maximum tolerated**	**Agent**	**Dose (mg/m**^**2**^**)**	
A	275	140	Gemcitabine	800	iv days 1 and 8 q 3 weekly
B	400	<200	Docetaxel	60	iv day 1 q 3 weekly
C	200	770	Docetaxel + G-CSF	60	iv day 1 q 3 weekly
D	770	≥770	Docetaxel + G-CSF	75	iv day 1 q 3 weekly

### MTDs, DLTs and recommended phase II dose

The MTDs, DLTs and recommended phase II dose are shown in Table 3. The MTD for PR-104 was 140 mg/m^2^ when combined with gemcitabine (Group A). Its DLT was grade four thrombocytopenia in two of three patients treated at the next highest dose-level of 275 mg/m^2^. Because its MTD was lower that the starting dose-level (275 mg/m^2^) and severe thrombocytopenia prohibited any dose-escalation, the PR-104-gemcitabine combination was not evaluated further.

**Table 3:** Dose escalation schemes and dose-limiting toxicities on cycle one

	**PR104 Dose**		**Patients**	**Dose-limiting toxicities**
	**Level**	**mg/m**^**2**^	**(n)**	
Group A PR104 + gemcitabine	1	275	3	Grade 4 thrombocytopenia (n=2)
	2	140	6	-
Group B PR104 + docetaxel 60	1	400	3	Grade 3 neutropenic fever (n=1)
	2	200	3	Grade 3 neutropenic fever (n=2)
Group C PR104 + docetaxel 60 + GCSF	1	200	3	-
	2	400	3	-
	3	550	3	-
	4	770	6	-
	5	1100	6	Grade 4 thrombocytopenia (=1) Grade 3 Fatigue (n=1)
Group D PR104 + docetaxel 75 + GCSF	1	770	6	-

The MTD for PR-104 was less than 200 mg/m^2^ when combined with docetaxel 60 mg/m^2^ (Group B). Its DLT was grade three neutropenic fever in one of three patients treated at the 400 mg/m^2^ dose-level and in two of three patients at the 200 mg/m^2^ dose-level. Because its MTD was lower than the starting dose-level (400 mg/m^2^) and severe (grade 3 and 4) neutropenia prohibited any dose-escalation, prophylactic G-CSF was added to the PR-104-docetaxel combination in the next combination group.

The MTD for PR-104 was 770 mg/m^2^ when combined with docetaxel 60 mg/m^2^ given with prophylactic G-CSF (Group C). Its DLT was grade four thrombocytopenia and grade three fatigue occurring in two of three patients treated at the next highest dose-level of 1100 mg/m^2^. The PR-104-doctaxel combination was explored further in the next combination group by increasing the dose of docetaxel to 75 mg/m^2^.

The MTD for PR-104 was greater than 770 mg/m^2^ when combined with docetaxel 75 mg/m^2^ given with prophylactic G-CSF (Group D). There was no DLT in a cohort of six patients treated at this dose-level in the combination group which was restricted to patients with one or no prior chemotherapy regimens. No further dose-escalation was undertaken because DLT had already been encountered at the next highest PR-104 dose-level (1100 mg/m^2^) in Group C who had received a lower dose of docetaxel than Group D.

The recommended phase II dose of PR-104 was 770 mg/m^2^ when combined with 60 or 75 mg/m^2^ of docetaxel both given on day one, with prophylactic G-CSF on day two, of a 21-day treatment cycle. No DLT occurred in a cohort of 12 patients treated at this dose-level.

### Other toxicities

Grade three or four toxicities associated with PR-104 combination treatment are shown in Tables 4 and 5. Haematological toxicity was the most common grade three or four toxicity and presented as thrombocytopenia, neutropenia with or without fever, anaemia or leukopenia. The most common non-haematological toxicity was fatigue, which was of grade three or four severity in twelve of 42 patients. Other non-haematological toxicity of grade three or four severity occurring in two or more patients included alopecia, respiratory infection, nausea and vomiting. Of 31 reported serious adverse events, 11 were considered related to the combinations of PR104 and gemcitabine or docetaxel +/− GCSF) and included: febrile neutropenia (n=4), neutropenic infection (n=2), respiratory infection (n=2), vomiting (n=1), dehydration (n=1) and hypersensitivity reaction (n=1). There were no treatment-related deaths reported during study treatment or within 30 days of the last dose of treatment administration.

**Table 4:** **Grade three or four treatment-related non-haematological adverse events by number of patients**^**1**^

**Adverse event**	**Group A**^**2**^**(n=9)**		**Group B**^**3**^**(n=6)**		**Group C**^**4**^**(n=21)**		**Group D**^**5**^**(n=6)**	
	**Gd 3**	**Gd 4**	**Gd 3**	**Gd 4**	**Gd 3**	**Gd 4**	**Gd 3**	**Gd 4**
Fatigue	2	-	-	-	6	-	2	2
Febrile neutropenia	-	3	2	-	1	-	-	-
Alopecia	-	-	-	-	3	-	-	-
Nausea	1	-	-	-	1	-	-	-
Respiratory infection	-	-	1	-	-	-	1	-
Vomiting	-	-	-	-	2	-	-	-
Anaemia	-	-	-	-	1	-	-	-
Anorexia	-	-	-	-	1	-	-	-
Dehydration	-	-	-	-	1	-	-	-
Diarrhoea	-	-	-	-	1	-	-	-
Leukopenia	1	-	-	-	-	-	-	-
QT/QTc prolongation	-	-	-	-	-	-	1	-
Weight loss	-	-	-	-	1	-	-	-

^1^Highest toxicity grade by CTCAE version 3.0 for each patient; ^2^ PR104 + gemcitabine; ^3^ PR104 + docetaxel 60; ^4^ PR104 + docetaxel 60 + GCSF; ^5^ PR104 + docetaxel 75 + GCSF.

**Table 5:** **Grade three or four treatment-related haematological adverse events by number of patients**^**1**^

**Adverse event**	**Group A**^**2**^**(n=9)**		**Group B**^**3**^**(n=6)**		**Group C**^**4**^**(n=21)**		**Group D**^**5**^**(n=6)**	
	**Gd 3**	**Gd 4**	**Gd 3**	**Gd 4**	**Gd 3**	**Gd 4**	**Gd 3**	**Gd 4**
Leucopenia	2	-	3	1	4	6	3	-
Neutropenia	2	1	3	1	3	6	2	1
Thrombocytopenia	-	3	-	-	2	4	-	2
Anaemia	1	-	-	-	3	-	1	-
Any haematological toxicity		4	3	1	5	7	1	3

^1^Highest toxicity grade by CTCAE version 3.0 for each patient; ^2^ PR104 + gemcitabine.

^3^ PR104 + docetaxel 60; ^4^ PR104 + docetaxel 60 + GCSF; ^5^ PR104 + docetaxel 75 + GCSF.

### Tumour response

Best tumour response was stable disease in 21 patients (50%), progressive disease in 15 patients (36%), partial response in four patients (10%) and not evaluable in two patients (5%). Of 21 patients with best tumour response of stable disease, 11 patients remained free of disease progression for at least three months and completed six or more cycles of study treatment. Of four patients with best tumour response of partial response, all four patients had received PR-104 at dose-levels of 770 mg/m^2^ or higher; two had partial responses confirmed on a subsequent scan (one each with nasopharyngeal carcinoma and non-small cell lung cancer), two had unconfirmed partial responses (one each with squamous cell of the tongue and nasopharyngeal carcinoma).

### Hypoxia imaging

A total of 13 patients underwent FMISO PET imaging at baseline and/or following two cycles of PR104 combination therapy. Pre-treatment hypoxia was detected in the tumours of seven patients, one each with melanoma, neuroendocrine carcinoma, non-small cell lung cancer, ovarian cancer, pancreatic cancer, colon cancer and small-cell lung cancer. Baseline FMISO PET scans were negative in four patients who had breast cancer, melanoma, pancreatic cancer and prostate cancer. Four of seven patients with positive FMISO PET scans for tumour hypoxia at baseline achieved best tumour response of stable disease that was maintained for a minimum of six treatment cycles or 18 weeks. None of the patients achieving partial tumour response had undergone FMISO PET imaging.

Six patients had FMISO PET imaging at both time-points allowing comparison of tumour hypoxia at baseline and again following two cycles of PR-104 combination therapy. Tumour hypoxia was present at both time-points in two patients, and not present at both time-points in a further two patients. One patient had tumour hypoxia present at baseline but not post-treatment and another had tumour hypoxia present post-treatment but not at baseline.

### Pharmacokinetics

**Figure 2:**
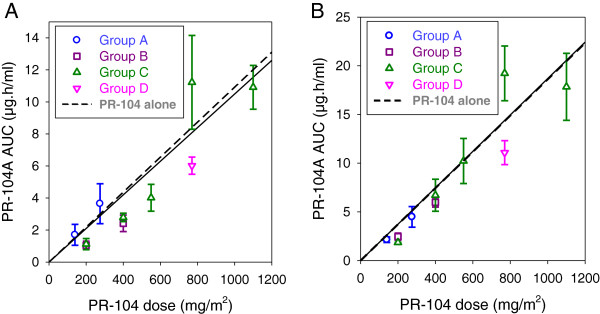
PR-104A plasma AUC versus PR-104 dose

**Figure 3:**
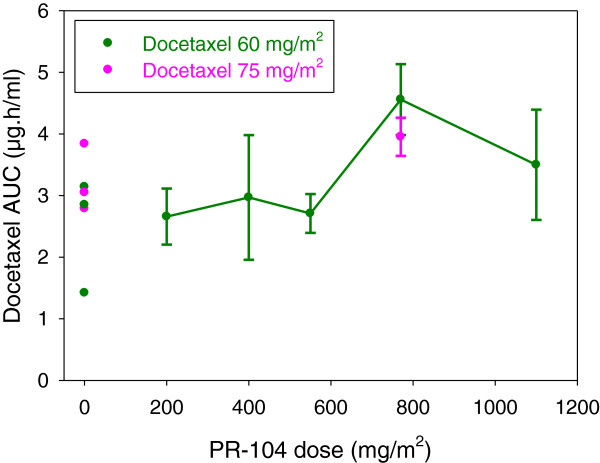
Docetaxel plasma AUC versus PR-104 dose

Pharmacokinetic studies of patients carried out on cycle one day one revealed plasma AUC values for PR-104 itself and PR-104A, the bioreductive prodrug generated from PR-104, that fell close to the AUC values reported in published studies of PR-104 given alone [7,8] when these values were plotted as a function of PR-104 dose-level (Figure 2). PR-104 dose-level corrected AUC values for the downstream metabolites from PR-104A (PR-104G, PR-104H, PR-104M and PR-104S1) also appeared similar across different PR-104 dose-levels and combination regimens (Table 6), as were terminal half-lives for PR-104A and its metabolites (data not shown) which were all approximately 0.5-1 h as previously reported [8,17,18]. Docetaxel plasma AUC values on cycle one day one of PR-104-docetaxel combination treatment, were similar at different PR-104 dose-levels and to those values reported in published studies of docetaxel given alone [19-21] (Figure 3). Cmax, AUC and t_1/2_ values for gemcitabine and its major inactive metabolite difluorodeoxyuridine (Table 7) appeared similar to published studies of gemcitabine alone at comparable dose-levels [22,23]. 

**Table 6:** Pharmacokinetic parameters of PR104 metabolites

	**PR104 Dose**		**Patients**	AUC/PR-104 dose (100*h.m^**2**^**.L**^**-1**^**)**^**a**^			
	**Level**	**mg/m**^**2**^	**(n)**	**PR104G**	**PR104H**	**PR-104M**	**PR-104S1**
Group A PR104 + gemcitabine	1	140	3	1.51 ± 0.63	0.12 ± 0.02	0.022±0.002	0.021±0.006
	2	275	6	1.18 ± 0.30	0.16 ± 0.03	0.015±0.002	0.020±0.006
Group B PR104 + docetaxel 60	1	200	3	0.86 ± 0.29	0.20 ± 0.06	0.016±0.003	0.010±0.003
	2	400	3	1.63 ± 0.73	0.12 ± 0.03	0.016±0.002	0.042±0.022
Group C PR104 + docetaxel 60 + GCSF	1	200	3	0.47 ± 0.26	0.07 ± 0.01	0.021±0.003	0.022±0.004
	2	400	3	1.75 ± 0.57	0.11 ± 0.03	0.012±0.001	0.017±0.002
	3	550	3	1.56 ± 0.38	0.16 ± 0.04	0.025±0.007	0.045±0.012
	4	770	6	2.56 ± 0.67	0.07 ± 0.01	0.012±0.002	0.062±0.019
	5	1100	6	2.76 ± 1.68	0.09 ± 0.01	0.010±0.001	0.019±0.030
Group D PR104 + docetaxel 75 + GCSF	1	770	6	1.16 ± 0.18	0.035 ± 0.005	0.009±0.001	0.022±0.006
Historical data for PR104 alone [8,16]	n/a	675	7	1.02 ± 0.24	0.06 ± 0.01	0.011 ± 0.002	0.042 ± 0.015
		1100	10	2.35 ± 0.85	0.09 ± 0.01	0.015± 0.002	0.041 ± 0.009

**Table 7:** **Gemcitabine and difluorodeoxyuridine plasma Cmax, AUC and half life in patients (n=7) given gemcitabine 800 mg/m**^**2**^**with PR-104. (Mean ± standard deviation)**

	**Cmax (mg/L)**	AUC (mg/L*hr)	**t**_**1/2**_**(hr)**
Gemcitabine	20.8 ± 10.6	4.87 ± 1.69	0.21 ± 0.06
Difluorodeoxyuridine	30.8 ± 9.56	219 ± 57.6	12.2 ± 2.34

^a^ Values and mean ± SEM.

## Discussion

This study established a recommended phase II dose for PR-104 of 770 mg/m^2^ for combination with standard clinical doses of docetaxel (60 to 75 mg/m^2^) [10], both given on day one with prophylactic G-CSF on day two of a three-week treatment cycle. At the recommended phase II dose, PR-104-docetaxel-G-CSF combination therapy appeared to be adequately tolerated without any DLT in a cohort of 12 patients treated at this dose-level. In contrast, two of three patients experienced DLT on cycle one of PR-104-docetaxel-G-CSF combination treatment at the next highest dose-level above the recommended phase II dose (PR-104 1100 mg/m^2^). At the recommended phase II dose-level, clinical benefit was apparent in five of a cohort of 12 patients either as partial tumour responses in three patients or as stable disease maintained for at least six treatment cycles (18 weeks) in a further two patients. It was not possible from our study to determine what contribution PR-104 may have made to this apparent clinical benefit relative to that made by docetaxel. However, the current study has provided a starting dose for phase II trials to address whether PR-104 enhances the therapeutic efficacy of docetaxel and to further evaluate the clinical safety of PR-104-docetaxel-G-CSF combination therapy.

Our study also found that combining PR-104 with gemcitabine or docetaxel in the absence of prophylactic G-CSF was not clinically feasible due to severe myelotoxicity. With the PR-104-gemcitabine combination, dose-limiting thrombocytopenia was encountered in two of three patients at the starting dose-level (275 mg/m^2^). Prophylactic G-CSF was not added to the PR-104-gemcitabine study group and the combination was not evaluated further due to thrombocytopenia being dose-limiting. With the PR-104-docetaxel combination given without G-CSF, dose-limiting febrile neutropenia occurred in three of six patients treated at or below the starting dose-level (400 mg/m^2^). The PR104-docetaxel combination was evaluated further with the addition of prophylactic G-CSF as dose-limiting neutropenia prohibited dose escalation of PR-104 without haematological growth factor support.

The unexpectedly severe and dose-limiting myelotoxicity encountered in this trial of PR-104-based combination chemotherapy appeared to have been due to a pharmacodynamic interaction, rather than a pharmacokinetic interaction, between PR-104 and gemcitabine or docetaxel. The plasma pharmacokinetics of docetaxel, gemcitabine, its major metabolite (difluorodeoxyuridine) and PR-104 and its metabolites (PR-104A, PR-104G, PR-104H, PR-104M and PR-104S1) determined in patients treated with PR-104 combined with gemcitabine or docetaxel appeared similar to published reports of the pharmacokinetics of PR-104 [7,8], gemcitabine [22,23] or docetaxel [19-21] given alone. Gemcitabine and docetaxel are known to cause blood cytopenias as single agents at standard clinical doses, presumably due to their antiproliferative effects against bone marrow blood progenitor cells occurring as a result of their main pharmacological actions that involve the inhibition of DNA synthesis in S-phase cells and disruption of microtubule assembly in the mitotic spindle of M-phase cells, respectively [9,10]. PR-104 is also myelotoxic as a single agent in clinical trials [7,8], via mechanisms that are currently unclear but that may involve its metabolic activation by hypoxia or AKR1C3 in normal bone marrow (personal communications, J. Down and K. Parmar) followed by DNA cross-linking and cytotoxicity to blood progenitor cells by mechanisms analogous to other myelosuppressive nitrogen mustards [24]. These considerations point to the possibility of the unexpectedly severe and dose-limiting blood cytopenias of the PR-104-chemotherapy combinations evaluated in this trial having been due to the overlapping myelotoxicity of PR-104, docetaxel and gemcitabine.

Dose-limiting myelotoxicity may have restricted PR-104 dose-escalation and PR-104A systemic exposure in this clinical study. When PR-104 was combined with gemcitabine or docetaxel without prophylactic G-CSF, for example, the MTD for PR-104 was less than 200 mg/m^2^ and PR-104A AUC values were ≤ 3 μg*hr/ml. Addition of prophylactic G-CSF to the combination of PR-104 and docetaxel 60 to 75 mg/m^2^ permitted escalation of PR-104 to a dose of 770 mg/m^2^ that achieved PR-104A AUC values ranging from about 5 to 20 μg*hr/ml. These PR-104A AUC values achieved at the recommended phase II dose for the PR-104-docetaxel-G-CSF combination were similar to the values from earlier clinical pharmacokinetic studies of PR-104 at this dose-level but lower than those achieved at its single agent MTD in phase Ia studies [7,8]. In contrast, mice appear to be able to tolerate both higher PR-104A systemic exposure, in the order of 50 μg*hr/ml [25], and the combination of PR-104 with gemcitabine or docetaxel at doses that achieved significant preclinical antitumour activity [5]. Furthermore, in human tumour xenograft murine models, the therapeutic antitumour activity of PR-104 alone or in combination with gemcitabine or docetaxel appears to be associated with doses [5] that achieve systemic exposures to PR-104A that were higher than those achieved by most patients treated in the current clinical study.

This was one of the first studies that we are aware of that incorporated hypoxia imaging with F MISO or an equivalent PET imaging agent into a multicentre early-phase trial of a novel chemotherapy combination in a broad oncology patient population. Previously, hypoxia imaging in therapeutic trials has been limited to only a few studies of mainly chemo-radiation protocols that were often restricted to head and neck cancer [26]. Several issues related to our experience are worthy of comment. Firstly, hypoxia imaging was successfully carried out in less than half of the study patients at baseline and very few scans were obtained post-treatment, pointing to issues with its feasibility in the context of multicentre early-phase oncology trials. Tumour hypoxia was detected in most but not all subjects undergoing FMISO scans and across a range of tumour types, suggesting that tumour hypoxia is very common among a broad phase I oncology patient population and unrestricted to any particular tumour site or specific histopathological diagnosis. No correlation was apparent in our study between the presence or absence of tumour hypoxia at baseline and subsequent tumour response to PR-104-based combination chemotherapy. However, the small sample size, heterogeneous patient population, range of PR-104 dose-levels and incomplete data-set for FMISO scans in our study may have contributed to the apparent lack of correlation between tumour hypoxia and therapeutic outcome from PR-104-combination chemotherapy. Further studies of hypoxia imaging in early-phase multicentre oncology trials are required to further evaluate its predictive value and feasibility.

It is interesting to compare the results of the current study with those recently reported for TH-302 [27-29], a hypoxia-activated prodrug of the cytotoxin bromoisophosphoramide mustard. Like PR-104, the mechanism of action of TH-302 involves its activation in hypoxia, via NADPH-cytochrome P450 oxidoreductases and other one-electron reductases, into reactive mustard species that crosslink DNA. In phase Ib and II clinical trials, the dose-limiting toxicities of TH-302 combined with gemcitabine and docetaxel primarily involved the haematological system, similar to PR-104. However, combining TH-302 with docetaxel and gemcitabine appeared not to require G-CSF support and increased tumour response rates and progression free survival, particularly in patients with advanced pancreatic cancer. These findings are very promising and further investigations of TH-302 and other hypoxia-activated prodrugs combined with conventional chemotherapy are awaited with interest.

## Conclusions

This phase Ib study has identified 770mg/m^2^ of PR-104 in combination with 60 to 75 mg/m^2^ of docetaxel administered on day one of a 21-day cycle with prophylactic G-CSF as the recommended dose and schedule for phase II studies. Combination of PR-104 with docetaxel results in dose-limiting and severe neutropenia that necessitates use of prophylactic G-CSF and further careful safety evaluation in phase II trials. Potential oncology indications for phase II trials of PR-104-docetaxel-G-CSF combination therapy include those tumour types for which docetaxel is already approved, such as breast, head and neck, non-small cell lung and prostate cancer. A phase II study of this treatment combination in patients with locally advanced or metastatic non-small cell lung cancer has been initiated (NCT00544674).

## Competing interests

WRW is a stock holder and advisor to Proacta, Inc. TJM is an employee of Proacta, Inc. The authors have no other competing interests to declare.

## Authors' contributions

MJM, MBJ, RKR, JR and NST contributed to the study design, patient recruitment, clinical study procedures, data interpretation and preparation of the final manuscript. YG and WRW contributed to the pharmacokinetic study procedures, data interpretation and preparation of the final manuscript. TJM contributed to the study design, data interpretation and preparation of the final manuscript. All authors read and approved the final manuscript.

## Pre-publication history

The pre-publication history for this paper can be accessed here:

http://www.biomedcentral.com/1471-2407/12/496/prepub

## References

[B1] Wilson WR Hay MP Targeting hypoxia in cancer therapy Nat Rev Canc 2011 11 6 393 410 10.1038/nrc3064 21606941

[B2] Toustrup K Sorensen BS Nordsmark M Busk M Wiuf C Alsner J Overgaard J Development of a hypoxia gene expression classifier with predictive impact for hypoxic modification of radiotherapy in head and neck cancer Canc Res 2011 71 17 5923 5931 10.1158/0008-5472.CAN-11-1182 21846821

[B3] Guise CP Abbattista MR Singleton RS Holford SD Connolly J Dachs GU Fox SB Pollock R Harvey J Guilford P The bioreductive prodrug PR-104A is activated under aerobic conditions by human aldo-keto reductase 1C3 Canc Res 2010 70 4 1573 1584 10.1158/0008-5472.CAN-09-3237 20145130

[B4] Singleton RS Guise CP Ferry DM Pullen SM Dorie MJ Brown JM Patterson AV Wilson WR DNA cross-links in human tumor cells exposed to the prodrug PR-104A: Relationships to hypoxia, bioreductive metabolism and cytotoxicity Canc Res 2009 69 9 3884 3891 10.1158/0008-5472.CAN-08-4023 19366798

[B5] Patterson AV Ferry DM Edmunds SJ Gu Y Singleton RS Patel K Pullen SM Hicks KO Syddall SP Atwell GJ Mechanism of action and preclinical antitumor activity of the novel hypoxia-activated DNA cross-linking agent PR-104 Clin Canc Res 2007 13 13 3922 3932 10.1158/1078-0432.CCR-07-0478 17606726

[B6] Guise CP Abbattista MR Tipparaju SR Lambie NK Su JC Li D Wilson WR Dachs GU Patterson AV Diflavin oxidoreductases activate the bioreductive prodrug PR-104A under hypoxia Mol Pharmacol 2012 81 1 31 40 10.1124/mol.111.073759 21984255

[B7] Jameson MB Rischin D Pegram M Gutheil J Patterson AV Denny WA Wilson WR A phase I trial of PR-104, a nitrogen mustard prodrug activated by both hypoxia and aldo-keto reductase 1C3, in patients with solid tumors Canc Chemother Pharmacol 2010 65 4 791 801 10.1007/s00280-009-1188-1 20012293

[B8] McKeage MJ Gu Y Wilson WR Hill A Amies K Melink TJ Jameson MB A phase I trial of PR-104, a pre-prodrug of the bioreductive prodrug PR-104A, given weekly to solid tumour patients BMC Cancer 2011 11 1 432 10.1186/1471-2407-11-432 21982454 PMC3205073

[B9] Noble S Goa KL Gemcitabine - a review of its pharmacology and clinical potential in non-small cell lung cancer and pancreatic cancer Drugs 1997 54 3 447 472 10.2165/00003495-199754030-00009 9279506

[B10] Sanofi-Aventis US Taxotere (docetaxel) full prescribing information 2011 Bridgewater NJ, Bridgewater NJ 08807 http://www.accessdata.fda.gov/drugsatfda_docs/label/2011/020449s064lbl.pdf

[B11] Jang SH Wientjes MG Au JLS Determinants of paclitaxel uptake, accumulation and retention in solid tumors Invest New Drugs 2001 19 2 113 123 10.1023/A:1010662413174 11392446

[B12] Huxham LA Kyle AH Baker JHE Nykilchuk LK Minchinton AI Microregional effects of gemcitabine in HCT-116 xenografts Canc Res 2004 64 18 6537 6541 10.1158/0008-5472.CAN-04-0986 15374965

[B13] Chapman JD Engelhardt EL Stobbe CC Schneider RF Gerald GE Measuring hypoxia and predicting tumor radioresistance with nuclear medicine assays Radiother Oncol 1998 46 3 229 237 10.1016/S0167-8140(97)00186-2 9572615

[B14] Therasse P Arbuck SG Eisenhauer EA Wanders J Kaplan RS Rubinstein L Verweij J Van Glabbeke M van Oosterom AT Christian MC New guidelines to evaluate the response to treatment in solid Tumors J Natl Canc Inst 2000 92 3 205 216 10.1093/jnci/92.3.205 10655437

[B15] Gu Y Wilson WR Rapid and sensitive ultra-high-pressure liquid chromatography-tandem mass spectrometry analysis of the novel anticancer agent PR-104 and its major metabolites in human plasma: application to a pharmacokinetic study J Chromatogr B Analyt Technol Biomed Life Sci 2009 877 27 3181 3186 10.1016/j.jchromb.2009.08.009 19709934

[B16] Atwell GJ Denny WA Synthesis of H-3- and H-2(4)-labelled versions of the hypoxia-activated pre-prodrug 2-((2-bromoethyl)-2,4-dinitro-6-(((2-(phosphonooxy)ethyl)amino)carbonyl) anilino)ethyl methanesulfonate (PR-104) J Label Compd Radiopharm 2007 50 1–2 7 12

[B17] Gu Y Atwell GJ Wilson WR Metabolism and excretion of the novel bioreductive prodrug PR-104 in mice, rats, dogs, and humans Drug Metab Dispos 2010 38 3 498 508 10.1124/dmd.109.030973 20019245

[B18] Gu Y Tingle MD Wilson WR Glucuronidation of anticancer prodrug PR-104A: species differences, identification of human UDP-Glucuronosyltransferases, and implications for therapy J Pharmacol Exp Ther 2011 337 3 692 702 10.1124/jpet.111.180703 21427202

[B19] Petrylak DP Macarthur RB O'Connor J Shelton G Judge T Balog J Pfaff C Bagiella E Heitjan D Fine R Phase I trial of docetaxel with estramustine in androgen-independent prostate cancer J Clin Oncol 1999 17 3 958 967 10071290 10.1200/JCO.1999.17.3.958

[B20] Baker SD Zhao M Lee CKK Verweij J Zabelina Y Brahmer JR Wolff AC Sparreboom A Carducci MA Comparative pharmacokinetics of weekly and every-three-weeks docetaxel Clin Canc Res 2004 10 6 1976 1983 10.1158/1078-0432.CCR-0842-03 15041715

[B21] Extra JM Rousseau F Bruno R Clavel M Lebail N Marty M Phase-I and pharmacokinetic study of Taxotere(RP-56976, NSC-628503) given as a short intravenous-infusion Canc Res 1993 53 5 1037 1042 8094996

[B22] Abbruzzese JL Grunewald R Weeks EA Gravel D Adams T Nowak B Mineishi S Tarassoff P Satterlee W Raber MN A phase-I clinical, plasma and cellular pharmacology study of gemcitabine J Clin Oncol 1991 9 3 491 498 1999720 10.1200/JCO.1991.9.3.491

[B23] Fogli S Danesi R Braud FD Pas TD Curigliano G Giovannetti E Del Tacca M Drug distribution and pharmacokinetic/pharmacodynamic relationship of paclitaxel and gemcitabine in patients with non-small-cell lung cancer Ann Oncol 2001 12 11 1553 1559 10.1023/A:1013133415945 11822754

[B24] Hall AG Tilby MJ Mechanisms of action of, and modes of resistance to, alkylating-agents used in the treatment of hematological malignancies Blood Rev 1992 6 3 163 173 10.1016/0268-960X(92)90028-O 1422285

[B25] Patel K Choy SSF Hicks KO Melink TJ Holford NHG Wilson WR A combined pharmacokinetic model for the hypoxia-targeted prodrug PR-104A in humans, dogs, rats and mice predicts species differences in clearance and toxicity Canc Chemother Pharmacol 2011 67 5 1145 1155 10.1007/s00280-010-1412-z 20683596

[B26] Rischin D Peters L Hicks R Hughes P Fisher R Hart R Sexton M D'Costa I von Roemeling R Phase I trial of concurrent tirapazamine, cisplatin, and radiotherapy in patients with advanced head and neck cancer J Clin Oncol 2001 19 2 535 542 11208848 10.1200/JCO.2001.19.2.535

[B27] Borad M Mita A Infante J Chiorean EG Vlahovic G Armstrong A Traynor AM Schelman W Langmuir V Eng C Complete phase Ib study of TH-302 in combination with gemcitabine (G), docetaxel (D) or pemetrexed (P) Ann Oncol 2010 21 172 172 10.1093/annonc/mdq203

[B28] Ryan D Reddy S Bahary N Uronis H Sigal DS Cohn A Schelman WR Chiorean EG Rosen PJ Ulrich B Phase II study of gemcitabine + TH-302 vs gemcitabine alone in patients with locally advanced and metastatic pancreatic cancer Ann Oncol 2012 23 8 8

[B29] Weiss GJ Infante JR Chiorean EG Borad MJ Bendell JC Molina JR Tibes R Ramanathan RK Lewandowski K Jones SF Phase 1 Study of the Safety, Tolerability, and Pharmacokinetics of TH-302, a Hypoxia-Activated Prodrug, in Patients with Advanced Solid Malignancies Clin Canc Res 2011 17 9 2997 3004 10.1158/1078-0432.CCR-10-3425 21415214

